# 1-Palmitoyl-2-Linoleoyl-3-Acetyl-*rac*-Glycerol Attenuates Streptozotocin-Induced Pancreatic Beta Cell Damage by Promoting Glucose Transporter 2 Endocytosis

**DOI:** 10.1128/MCB.00157-19

**Published:** 2019-10-11

**Authors:** Jimin Kim, Joo Heon Kim, Ki-Young Sohn, Sun Young Yoon, Jae Wha Kim

**Affiliations:** a Cell Factory Research Center, Division of Systems Biology and Bioengineering, Korea Research Institute of Bioscience and Biotechnology, Daejeon, Republic of Korea; b Department of Functional Genomics, KRIBB School of Bioscience, University of Science and Technology, Daejeon, Republic of Korea; c Department of Pathology, EulJi University School of Medicine, Daejeon, Republic of Korea; d Division of Global New Drug Development, Enzychem Lifesciences, Jecheon, Republic of Korea

**Keywords:** PLAG, streptozotocin, beta cell, GLUT2, endocytosis

## Abstract

Streptozotocin (STZ) is widely used to induce diabetic rodent models. It is specifically toxic to pancreatic beta cells and causes severe destruction and dysfunction. We investigated the effect of 1-palmitoyl-2-linoleoyl-3-acetyl-*rac*-glycerol (PLAG) on an STZ-induced diabetic mouse model. PLAG attenuated the glucose increase and maintained serum insulin at levels similar to those seen with control mice. In pancreatic beta cell line INS-1, STZ-induced cell apoptosis and intracellular reactive oxygen species (ROS) generation were significantly reduced to nearly normal levels after PLAG treatment. Glucose transporter 2 (GLUT2) localization analyses and glucose uptake assays showed that PLAG accelerated GLUT2 internalization, which ameliorated excessive entry of glucose, as well as STZ. STZ-induced cytotoxic effects were significantly reduced in PLAG-treated groups. The biological activity of PLAG was further confirmed in GLUT2-silenced cells, and the specificity of PLAG was verified using its derivative 1-palmitoyl-2-linoleoyl-3-hydroxyl-*rac*-glycerol (PLH). Our results suggest that PLAG may be a useful agent for protecting beta cells in the setting of excessive glucose influx.

## Abstract (precis)

Streptozotocin (STZ) is widely used to induce diabetic rodent models. It is specifically toxic to pancreatic beta cells and causes severe destruction and dysfunction. We investigated the effect of 1-palmitoyl-2-linoleoyl-3-acetyl-*rac*-glycerol (PLAG) on an STZ-induced diabetic mouse model. PLAG attenuated the glucose increase and maintained serum insulin at levels similar to those seen with control mice.

## INTRODUCTION

Streptozotocin (STZ) was isolated from a strain of the soil microbe Streptomyces achromogenes and was initially discovered as an antibiotic (1). STZ has specific toxic effects on insulin-releasing pancreatic beta cells, making it a useful diabetogenic agent in various rodent models (2). STZ induces beta cell destruction through a variety of diabetogenic mechanisms. STZ causes DNA fragmentation and alkylation of cellular components, eventually leading to cell death (3). Damaged DNA requires activation of the NAD-dependent enzyme poly(ADP-ribose) polymerase (PARP), which results in NAD^+^ depletion and decreased ATP concentrations that inhibit insulin synthesis and secretion by pancreatic beta cells (4, 5). *O*-GlcNAcase is a glycoside hydrolase, and its inhibition by STZ causes cytosolic accumulation of harmful proteins and leads to cell apoptosis (6, 7). The nitrosourea group in STZ acts as an intracellular nitric oxide (NO) donor, and STZ generates free radicals and intracellular reactive oxygen species (ROS) that ultimately cause oxidative stress (8). Oxidative stress is also caused by glucose auto-oxidation, formation of advanced glycation products, and protein glycation under STZ-induced diabetic conditions (9, 10). Because pancreatic beta cells express low levels of antioxidant enzymes, making them vulnerable to oxidative stress, STZ-induced oxidative stress leads to beta cell apoptosis (11). Indeed, NRF2 activators that increase antioxidant enzyme expression showed outstanding cytoprotective effects in various diabetic models (12–14). Elevated blood glucose levels induce inflammatory cell recruitment, and these cells secrete cytokines and activate stress signal pathways (15, 16). All of these toxic effects of STZ suppress pancreatic beta cell function and destroy the cells.

Glucose transporters (GLUTs) transport glucose across the cell membrane and include at least 14 types with very similar structures; they are differentially expressed in various cell types and play a role in glucose metabolism (17). STZ can access cells only through GLUT2, so it induces the most damage in pancreatic beta cells that highly express GLUT2; however, it also impairs liver and kidney cells (18, 19). Because GLUT2-dependent glucose uptake occurs in beta cells, its expression is necessary for insulin secretion in response to glucose sensing (20, 21). Unlike GLUT4, which is predominantly expressed in adipocytes and muscle cells and is present in cytoplasmic vesicles prior to translocation to the plasma membrane following insulin stimulation, GLUT2 is largely found on the cell membrane (22). Many studies have reported that chronic diabetic conditions result in loss of GLUT2 expression in pancreatic beta cells (23, 24), but the exact mechanisms involved have not been elucidated. Several recent studies suggested that N-linked glycosylation of GLUT2 is the cause of cell surface level reduction (25) and that GLUT2 endocytosis occurs at high extracellular glucose concentrations (26). Another group found that the endosomal protein Wiskott-Aldrich syndrome homologue (WASH) regulates GLUT2 trafficking and glucose homeostasis in pancreatic beta cells (27).

1-Palmitoyl-2-linoleoyl-3-acetyl-*rac*-glycerol (PLAG) is a monoacetyldiglyceride, originally isolated from the antlers of Sika deer, that is chemically synthesized as a single compound (28, 29). PLAG showed efficacy as a potential therapeutic agent in various disease models such as asthma (30, 31), arthritis (32), hepatitis (33), lung inflammation (34), and oral mucositis (35). PLAG also inhibited biliary cancer cell metastasis (28) and alleviated chemotherapy-induced neutropenia (36, 37) and thrombocytopenia (38). In this study, we investigated the biological effect of PLAG on STZ-induced pancreatic cell damage. PLAG significantly attenuated the cytotoxic actions of STZ by accelerating GLUT2 internalization and then promoting its return to the membrane. Collectively, PLAG has a protective effect by promoting GLUT2 intracellular trafficking, which helps attenuate ROS generation and decrease cell apoptosis in an STZ-induced diabetic model.

## RESULTS

### PLAG attenuated STZ-induced diabetic symptoms in the STZ mouse model.

**FIG 1:**
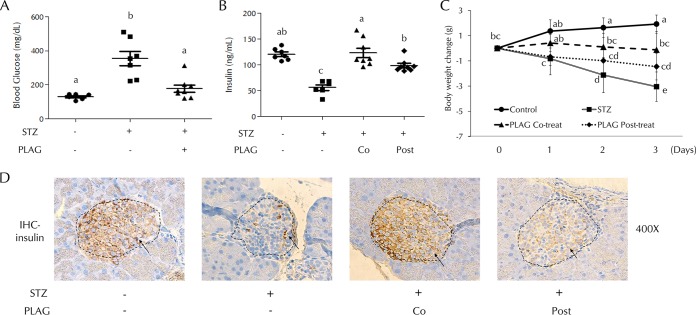
Effect of PLAG on STZ-injected mice. (A) Blood glucose levels were measured on day 1. (B) Serum insulin levels were measured on day 4. Co, cotreatment; Post, posttreatment. (C) Body weight changes were measured from days 0 to 3. Control group, *n* = 7; STZ group, *n* = 7; PLAG cotreatment group, *n* = 8; PLAG posttreatment group, *n* = 8. Statistical significance was determined by ANOVA (Tukey’s test). ANOVA results are shown as letters above dot plots and graphs. Means not sharing the same letter are statistically significantly different. (D) Immunohistochemistry (IHC) for insulin in pancreatic islets of mice. All images represent ×400 magnification.

Mice were injected intraperitoneally with STZ (200 mg/kg body weight) and treated with PLAG (250 mg/kg, administered orally [p.o.]). Blood glucose levels were measured on day 1 (Fig. 1A), and an increase was observed in STZ-treated animals. However, the PLAG cotreatment group did not show a significant increase. Serum insulin levels were examined on day 4 (Fig. 1B). Insulin secretion was decreased in the STZ group, but the PLAG cotreatment group results were similar to those seen with the control animals. The levels of insulin secretion in the PLAG posttreatment group were similar to those seen with the control animals, even though PLAG was given beginning on day 1 when the blood glucose level was already increased by STZ treatment. Body weight changes were measured from day 0 to day 3 (Fig. 1C). Body weight steadily increased in the control group, the members of the PLAG cotreatment group mainly maintained their weight on day 0, and body weight was drastically decreased in the STZ group. Although the PLAG posttreatment animals showed decreased weight, the decrease was not as pronounced as that seen with the STZ group. We performed immunohistochemistry analyses to examine the levels of insulin expression in beta cell islets (Fig. 1D, dotted lines). The control and PLAG cotreatment groups had many beta cells expressing insulin, but the islets in the STZ group were slightly shrunken and insulin expression was reduced. The shape of islets in the PLAG posttreatment group was normal, but the level of insulin expression was somewhat lower than that seen in the control group but higher than that seen in the STZ group.

### PLAG reduced STZ-induced cell apoptosis.

**FIG 2:**
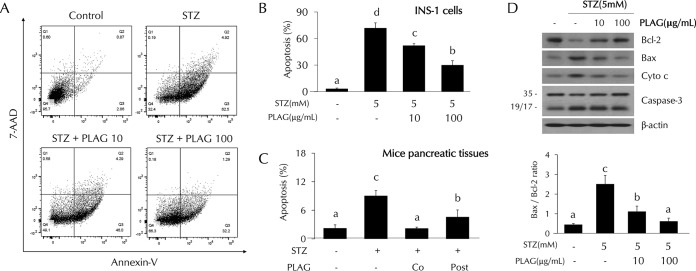
Effect of PLAG on STZ-induced cell apoptosis. (A to C) Cell apoptosis in INS-1 cells (A and B) and pancreatic tissues (C) of mice (on day 4) was analyzed by flow cytometry using annexin V and 7-AAD dyes. (D) (Top panel) Expression of apoptosis-related proteins such as Bcl-2, Bax, cytochrome *c*, and caspase-3 was analyzed by Western blotting. (Bottom panel) Bax/Bcl-2 ratios are represented by a bar graph. Statistical significance was determined by ANOVA (Tukey’s test). ANOVA results are shown as letters above columns. Means not sharing the same letter are statistically significantly different.

The effect of PLAG on STZ-induced cell apoptosis was analyzed using flow cytometry. Cell apoptosis was increased up to about 70% from baseline in STZ-treated INS-1 cells. The level of apoptosis observed in the cells treated with 10 μg/ml of PLAG was ∼50%, and it was ∼30% in the 100 μg/ml PLAG-treatment group, indicating dose-dependent protection (Fig. 2A and B). PLAG also showed a protective effect with respect to STZ-induced cell apoptosis in pancreatic tissues of mice (Fig. 2C). Additionally, apoptosis-related proteins were analyzed by Western blotting (Fig. 2D). Levels of antiapoptotic protein BCL-2 (B-cell lymphoma 2) were decreased by STZ treatment and recovered by PLAG treatment. In contrast, expression of apoptosis-related proteins BAX (BCL-2 associated X), cytochrome *c*, and caspase-3 were increased by STZ treatment and attenuated by PLAG addition.

### PLAG accelerated GLUT2 endocytosis and then promoted its membrane return in INS-1 cells.

**FIG 3:**
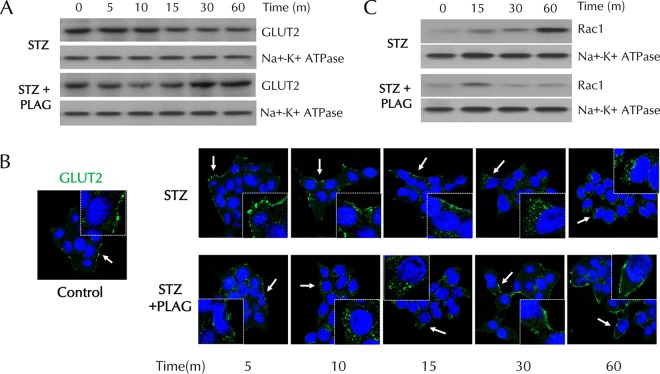
Effect of PLAG on plasma membrane GLUT2 expression in INS-1 cells. (A and B) GLUT2 expression in membrane fractions was analyzed by Western blotting (A) and observed with confocal microscopy (B). Arrows indicate results of GLUT2 expression. (C) Rac1 expression in membrane fractions was analyzed by Western blotting.

To investigate the effect of PLAG on GLUT2 plasma membrane localization, membrane proteins were fractionated and analyzed by Western blotting (Fig. 3A). Levels of membrane-expressed GLUT2 steadily decreased in STZ-treated cells. In PLAG-treated cells, GLUT2 expression gradually decreased until 10 min and then recovered and returned to control levels at 60 min. We also assessed GLUT2 localization by immunofluorescence analysis (Fig. 3B). GLUT2 was present in the cell membrane under normal conditions and was not detected in internalized locations in STZ-treated cells. In PLAG-treated cells, accelerated GLUT2 internalization was observed (similarly to the Western blotting results), and GLUT2 was again observed in the membrane at 60 min. The expression of RAC1, an NADPH oxidase that produces ROS, steadily increased in membrane fractions in the STZ group but was attenuated in the PLAG-treated group after a slight increase in 15 min, the time at which GLUT2 endocytosis occurred (Fig. 3C).

### PLAG reduced STZ-induced intracellular ROS generation.

**FIG 4:**
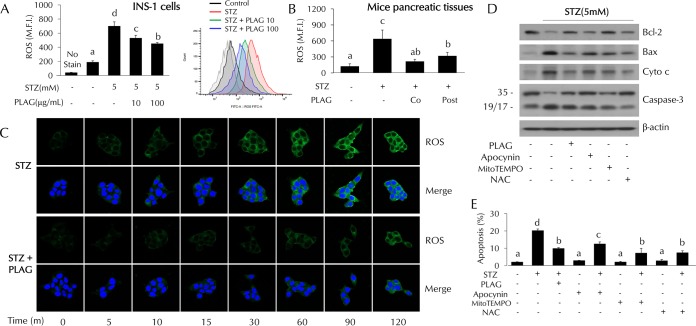
Effect of PLAG on intracellular ROS generation. (A and B) Intracellular ROS generation in INS-1 cells (A) and pancreatic tissues (B) of mice (on day 4) was analyzed by flow cytometry using DCFH-DA dye. M.F.I., mean fluorescence intensity. (C) ROS expression was observed with confocal microscopy. (D and E) Apoptosis-related protein expression (D) and cell apoptosis (E) were analyzed in ROS inhibitor-treated cells. Statistical significance was determined by ANOVA (Tukey’s test). ANOVA results are shown as letters above columns. Means not sharing the same letter are statistically significantly different.

Intracellular ROS levels were increased in the STZ-treated cells and dose dependently decreased in PLAG-treated INS-1 cells (Fig. 4A). ROS levels decreased by PLAG treatment were also observed in pancreatic tissues of mice (Fig. 4B). ROS levels rapidly increased in a time-dependent fashion in STZ-treated cells, but this was attenuated in PLAG-treated cells (Fig. 4C). We further examined cell apoptosis in cells cotreated with three types of ROS inhibitor to examine the association of intracellular ROS generation with pancreatic beta cell apoptosis (Fig. 4D and E). Apocynin (NADPH oxidase inhibitor), MitoTEMPO (mitochondrial ROS inhibitor), and *N*-acetyl-l-cysteine (NAC; an overall ROS inhibitor) were used. Significant reductions in the levels of expression of apoptosis-related proteins and STZ-induced cell apoptosis were observed in inhibitor-treated cells. These results suggest that ROS generation following STZ treatment contributes to pancreatic beta cell apoptosis.

### PLAG modulated precipitous glucose influxes.

**FIG 5:**
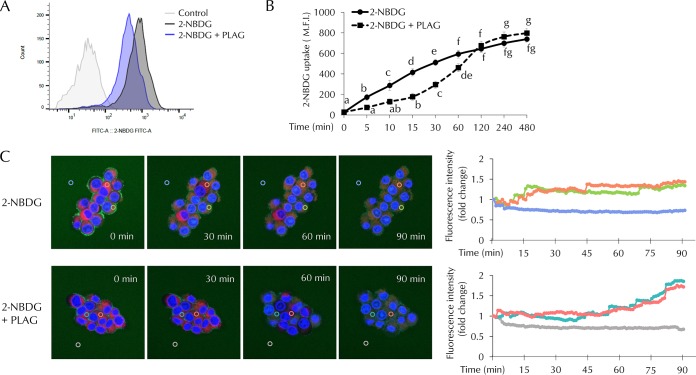
Effect of PLAG on beta cell glucose uptake. Glucose uptake was analyzed using 2-NBDG, a fluorescence-conjugated glucose analogue. (A) Intracellular 2-NBDG levels were measured 1 h after 2-NBDG treatment. FITC-A, fluorescein isothiocyanate A. (B) Intracellular 2-NBDG levels were measured for 8 h by flow cytometry. (C) Live-cell images of merged nucleus (Hoechst; blue), cytosol (Cell-tracker; red), and 2-NBDG (green) in INS-1 cells were captured using a Zeiss LSM800 microscope. Fluorescence intensities in regions of interest determined by continuous measurement are marked with circles. The zero time point level was normalized to a value of 1, and data representing fold change with respect to the zero time point are shown in line graphs. Statistical significance was determined by ANOVA (Tukey’s test). ANOVA results are shown as letters above graphs. Means not sharing the same letter are statistically significantly different.

Glucose uptake assays were performed using 2-[*N*-(7-nitrobenz-2-oxa-1,3-diazol-4-yl)amino]-2-deoxy-d-glucose (2-NBDG) conjugated with fluorescent dye. Intracellular 2-NBDG levels were measured at 60 min (Fig. 5A) and successively calculated at 5 min to 480 min after 2-NBDG treatment (Fig. 5B). Fluorescence intensity was measured in 2-NBDG-treated cells, but lower fluorescence intensity was detected in the PLAG-treated group at 60 min (Fig. 5A). As shown in Fig. 5B, glucose uptake was attenuated in PLAG-treated cells until 120 min. The results suggest that PLAG accelerated internalization of GLUT2, thus limiting glucose entry. However, when GLUT2 receptor returned to the membrane, accumulated glucose uptake among the members of the PLAG-treated group increased to a level similar to or higher than that seen with the 2-NBDG group after 120 min. We also observed intracellular 2-NBDG in live cell imaging using confocal microscopy (Fig. 5C). In the group treated only with 2-NBDG, 2-NBDG was observed at the cell membrane at 0 min and levels gradually increased in the cytoplasm, and fluorescence intensity increased in a time-dependent manner. Intracellular 2-NBDG levels increased more slowly in the PLAG-treated group. Additionally, the fluorescence intensity was determined by continuous measurement in regions of interest (marked with circles in Fig. 5C). The level measured at the zero time point was normalized to a value of 1, and fold change is shown with respect to the zero time point in line graphs. In the group treated only with 2-NBDG, the fluorescence intensity increased to the saturated level within approximately 45 min. On the other hand, it took a relatively long time to reach the highest level in the PLAG-treated group. These results suggest that PLAG may attenuate the deleterious effects of rapid glucose uptake through promoting GLUT2 endocytosis and simultaneously maintaining normal beta cell function.

### GLUT2 was essential for STZ-induced cellular damage.

**FIG 6:**
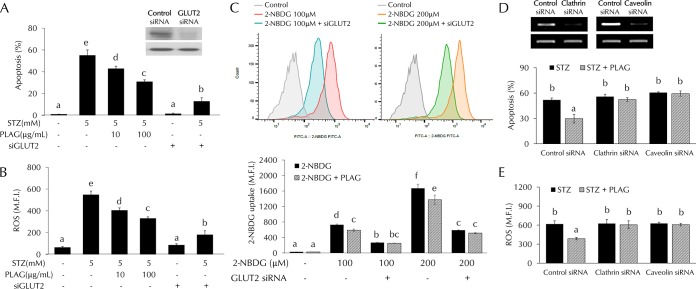
STZ effects in GLUT2-silenced cells. (A to C) Cell apoptosis (A), intracellular ROS generation (B), and glucose uptake (C) were analyzed by flow cytometry in GLUT2 siRNA-transfected cells. (D and E) Effect of PLAG on cell apoptosis (D) and ROS generation (E) in clathrin siRNA-transfected or caveolin siRNA-transfected cells. Statistical significance was determined by ANOVA (Tukey’s test). ANOVA results are shown as letters above columns. Means not sharing the same letter are statistically significantly different.

GLUT2-silenced cells were prepared to clarify the role of GLUT2 in STZ-treated cells. INS-1 cells were transfected with GLUT2 small interfering RNA (siRNA) to determine whether GLUT2 expression is related to STZ-induced cell apoptosis and ROS generation. Levels of cell apoptosis (Fig. 6A) and intracellular ROS generation (Fig. 6B) were noticeably increased in STZ-treated cells but not in GLUT2-silenced cells. Glucose uptake levels were also not significantly increased in GLUT2-silenced cells (Fig. 6C). To determine if PLAG biological activity was dependent on intracellular trafficking of GLUT2, we silenced the expression of the endocytosis-related proteins clathrin and caveolin using siRNAs (Fig. 6D and E). PLAG did not influence apoptosis or ROS generation in clathrin- or caveolin-silenced cells.

### Specificity of PLAG activity in STZ-treated pancreatic beta cells.

**FIG 7:**
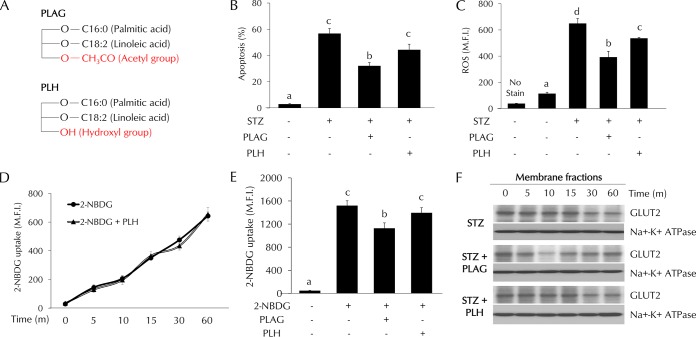
Comparison of the effects of PLAG and PLH in STZ-treated cells. (A) Simple structures of PLAG and PLH. (B to E) Cell apoptosis (B), intracellular ROS generation (C), and glucose uptake (D and E) were analyzed by flow cytometry. (F) GLUT2 expression in membrane fractions was analyzed by Western blotting. Statistical significance was determined by ANOVA (Tukey’s test). ANOVA results are shown as letters above columns. Means not sharing the same letter are statistically significantly different.

1-Palmitoyl-2-linoleoyl-3-hydroxyl-*rac*-glycerol (PLH) is a structural analogue of PLAG. The structures of PLAG and PLH are shown in Fig. 7A. PLAG has an acetyl group, but PLH has a hydroxyl group at position 3 of glycerol. We compared the effects of PLAG and PLH on STZ-treated cells. Cell apoptosis (Fig. 7B), ROS generation (Fig. 7C), and glucose uptake (Fig. 7D and E) were analyzed by flow cytometry. PLAG was more effective than PLH in decreasing cell apoptosis and ROS generation. In glucose uptake assays, the pattern shown by the PLH-treated group was similar to that shown by the control group. Because there was no effect on glucose uptake in PLH-treated cells, we examined GLUT2 expression in membrane fractions using Western blot analysis (Fig. 7F). PLAG treatment accelerated GLUT2 endocytosis at 10 min, prohibiting 2-NBDG uptake. In contrast, there was no change in the levels of 2-NBDG uptake or GLUT2 internalization in PLH-treated cells (Fig. 7D to F). These results confirm the specificity of PLAG in promoting GLUT2 endocytosis.

## DISCUSSION

Glucose is the body’s major source for energy metabolism; it is used in most cells and plays an important role in their function. After eating, blood glucose levels increase and glucose is absorbed into muscle or adipose tissues in response to insulin secreted from pancreas beta cells and is utilized as an energy source. However, elevated glucose levels have deleterious effects on pancreatic beta cell function and survival (39). High levels of glucose-induced beta cell overstimulation result in the inability of insulin synthesis to follow the rate of insulin secretion (40, 41). As a result, glucose-stimulated insulin secretion (GSIS), which is the most important function of beta cells, does not occur properly. High levels of glucose have detrimental effects on beta cell survival, inducing oxidative stress, endoplasmic reticulum stress (42), and apoptosis and inhibiting cell differentiation (43, 44). Modulation of excessive glucose uptake is therefore necessary to maintain pancreatic beta cell function and survival.

In this study, we found that PLAG accelerated GLUT2 internalization and then promoted its return to the membrane. As a result, PLAG-treated cells showed no evidence of rapid entry of extracellular STZ, resulting in attenuated pancreatic beta cell damage. The level of GLUT2 membrane expression was decreased for 15 min due to internalization promoted by PLAG (Fig. 3). Analysis of glucose uptake using fluorescence-conjugated 2-NBDG showed that glucose slowly entered cells at early time points after PLAG addition (Fig. 5). This modulation attenuated the STZ-induced generation of intracellular ROS and cell apoptosis. PLAG was able to protect beta cells via modulation of GLUT2 membrane localization for a few minutes, thus preventing a precipitous influx of glucose. GLUT2 returned to the membrane after a short period of internalization induced by PLAG, allowing glucose to enter cells at a constant concentration and enabling GSIS and function as normal beta cells.

The modulating effect of PLAG on GLUT2 internalization was further proven in endocytosis-related protein-silenced cells (Fig. 6). PLAG did not affect apoptosis or ROS generation in clathrin- or caveolin-silenced cells, indicating that PLAG promotes GLUT2 endocytosis. Additionally, the specificity of PLAG activity was verified through comparison with PLH, a lipid component with a structure similar to that of PLAG (Fig. 7). PLH did not have any effects on apoptosis, ROS generation, or glucose uptake in the STZ-induced diabetic model. These results suggest that the acetyl group at the third position of glycerol is responsible for the unique properties of PLAG.

In conclusion, we observed that reduced GLUT2 membrane expression could represent a protective mechanism rather than a result of exposure to the diabetic environment. Excessive glucose uptake induces ROS production and oxidative stress and must be controlled; this is made possible by PLAG promoting GLUT2 endocytosis. Even if beta cells are exposed to high glucose levels, PLAG can regulate the rapid influx of glucose to keep the cells from becoming overstimulated. Protected beta cells can normally secrete insulin, which allows blood glucose to be absorbed into adipose and muscle tissues. This may be why PLAG exerted a remarkable effect in the STZ-induced diabetic mouse model. Collectively, our results suggest that PLAG could be developed as an agent to protect against tissue damage due to diabetes-associated hyperglycemia.

## MATERIALS AND METHODS

### Cell culture.

INS-1 rat insulinoma pancreas beta cells were cultured in RPMI 1640 medium (Welgene, Gyeongsangbuk-do, Republic of Korea) containing 10% fetal bovine serum (Tissue Culture Biologicals, Long Beach, CA), 50 μM β-mercaptoethanol, 100 units/ml penicillin, and 100 μg/ml streptomycin (antibiotic-antimycotic solution; WelGENE). The cells were grown in a humidified atmosphere with 5% CO_2_ at 37°C.

### Chemicals and reagents.

PLAG was obtained from Enzychem Lifesciences (Jecheon, Republic of Korea). STZ was purchased from Enzo Life Sciences (Farmingdale, NY). PLAG was dissolved in ethanol for *in vitro* treatment, and the final working concentration was 0.1% (vol/vol). For *in vivo* experiments, PLAG was dissolved in phosphate-buffered saline (PBS); STZ was dissolved in 0.1 M citrate buffer (pH 4.5).

### Diabetic animal model.

Ten-week-old male BALB/c mice from Koatech (Gyeonggi-do, Republic of Korea) were obtained and divided into the following four groups (with seven to eight mice per group): control, STZ-only treatment, PLAG cotreatment, and PLAG posttreatment. After a 16-h fast, the three treated groups were injected intraperitoneally with STZ (200 mg/kg body weight) prepared fresh in citrate buffer. STZ mice received no additional treatment. On the same day, PLAG cotreatment group mice began treatment with PLAG (250 mg/kg, p.o.) once daily for 3 consecutive days. The PLAG-posttreatment group received PLAG (250 mg/kg p.o.) for 2 consecutive days beginning 1 day after STZ injection. Blood was collected via the retro-orbital plexus, and blood glucose levels were monitored during the experiment. Blood glucose was measured using an Accu-Chek glucometer (Roche, Seoul, Republic of Korea). All mice were sacrificed on day 4, and tissues were collected and fixed in 10% formalin for further analysis. All animal experiments were approved by the Institutional Animal Care and Use Committee of the Korea Research Institute of Bioscience and Biotechnology and were performed in compliance with the National Institute of Health Guidelines for the care and use of laboratory animals and Korean national laws for animal welfare.

### Enzyme-linked immunosorbent assay (ELISA).

Ninety-six-well microtiter plates were coated with anti-insulin antibody (ab8304; Abcam, Cambridge, United Kingdom) at 4°C overnight and then washed three times with PBS containing Tween 20 (PBST). Wells were blocked with 2% bovine serum albumin (BSA) at room temperature for 1 h, followed by the addition of samples. After incubation for 2 h, the plates were washed three times with PBST, horseradish peroxidase (HRP)-conjugated insulin antibody (ab28063; Abcam) was added, and the reaction mixture was incubated for 1 h. After three washes, 100 μl of tetramethylbenzidine (TMB) substrate solution was added to each well, and the reaction was terminated by adding 100 μl of 2 M sulfuric acid. Secreted insulin levels were measured using an EMax precision microplate reader (Molecular Devices, Sunnyvale, CA) at 450 nm.

### Pancreas islet histopathology.

Pancreas tissues were fixed in 10% formalin, embedded in paraffin, and divided into sections that were 4 μm thick. For immunohistochemistry, sections were deparaffinized and dehydrated using xylene and a graded ethanol series. Staining was performed using a Real EnVision detection system peroxidase–3,3′-diaminobenzidine (DAB) kit (Dako, Glostrup, Denmark) according to the manufacturer’s instructions, and the results were then observed under a light microscope (Olympus, Tokyo, Japan).

### Western blot analyses.

Cells were lysed by the use of radioimmunoprecipitation assay (RIPA) buffer (lipopolysaccharide [LPS] solution; Daejeon, South Korea) supplemented with protease and phosphatase inhibitors (Thermo Scientific, Waltham, MA). We then performed membrane protein fractionations using a Mem-PER Plus kit (Thermo Scientific) by following the manufacturer’s instructions. Proteins were separated on 12% sodium dodecyl sulfate-polyacrylamide gels and transferred to polyvinylidene difluoride membranes (EMD Millipore, Darmstadt, Germany). The membranes were blocked with 5% BSA for 1 h and incubated with primary antibodies to GLUT2 (bs-0351r; Bioss Antibodies, Woburn, MA), RAC1 (catalog no. 03589; EMD Millipore), BAX (catalog no. BS1030; Bioworld Tech, St. Louis Park, MN), BCL-2 (BS1031, Bioworld), cytochrome *c* (catalog no. 4272; Cell Signaling Technology, Danvers, MA), caspase-3 (catalog no. 9662; Cell Signaling Technology), and Na^+^-K^+^ ATPase (catalog no. 3010S; Cell Signaling Technology). After three washes in PBST, membranes were incubated with HRP-conjugated secondary antibodies (Enzo Life Sciences) (dilution, 1:5,000) for 1 h at room temperature. Protein bands were detected using ECL reagent (Thermo Scientific) and then visualized on films.

### Flow cytometry.

Cells were collected by trypsinization and washed with PBS. For cell apoptosis analyses, INS-1 cells were incubated with annexin V (BD Biosciences, Franklin Lakes, NJ) for 10 min at room temperature and were then stained with 7-aminoactinomycin D (7-AAD) (BD Biosciences). For intracellular ROS analyses, cells were incubated with 2 μM 2′,7′-dichlorodihydrofluorescein diacetate (DCFH-DA; Invitrogen, Carlsbad, CA) for 30 min at 37°C. The analysis was performed using a BD FACSVerse flow cytometer (BD Biosciences).

### Immunofluorescence assay.

Cells were grown on glass coverslips in 24-well plates and treated with STZ and PLAG. After washes were performed with ice-cold PBS, the cells were fixed with 4% formaldehyde. The cells were incubated with anti-GLUT2 antibodies (dilution, 1:500) and then stained with an Alexa Fluor 488-conjugated secondary antibody (Enzo Life Sciences) (dilution, 1:1,000) and DAPI (4′,6-diamidino-2-phenylindole; Invitrogen). For ROS analyses, cells were stained with DCFH-DA (Invitrogen) and DAPI (Invitrogen). Stained cells were observed under a Zeiss LSM800 confocal microscope (Carl Zeiss, Jena, Germany).

### Transfection.

Transfection with siRNAs was performed using HiPerFect reagent (Qiagen, Hilden, Germany) according to the protocol of the manufacturer. Specific siRNAs directed against GLUT2, clathrin, and caveolin were obtained from Santa Cruz Biotechnology (Dallas, TX).

### 2-NBDG uptake assay.

2-[*N*-(7-Nitrobenz-2-oxa-1,3-diazol-4-yl)amino]-2-deoxy-d-glucose (2-NBDG; Thermo Scientific catalog no. N13195) was used for glucose uptake assays. Serum-containing complete medium was removed, and INS-1 cells were washed in PBS. The cells were cultured in glucose-free culture media for 1 h at 37°C and were then treated with 2-NBDG. Intracellular fluorescence was measured using a BD FACSVerse flow cytometer (BD Biosciences).

### Statistical analyses.

Data are presented as means ± standard deviations. The statistical significance of differences between means was examined by one-way analysis of variance (ANOVA). *P* values of *<*0.05 were considered statistically significant.
